# Iterative heterogeneous graph learning for knowledge graph-based recommendation

**DOI:** 10.1038/s41598-023-33984-5

**Published:** 2023-04-28

**Authors:** Tieyuan Liu, Hongjie Shen, Liang Chang, Long Li, Jingjing Li

**Affiliations:** 1 School of Artificial Intelligence, Guilin University of Electronic Technology, Guilin, 541000 China; 2 Guangxi key Laboratory of Trusted Software, Guilin University of Electronic Technology, Guilin, 514000 China; 3 College of Cyber Security, Jinan University, Guangzhou, 510632 China

**Keywords:** Engineering, Electrical and electronic engineering

## Abstract

Incorporating knowledge graphs into recommendation systems has attracted wide attention in various fields recently. A Knowledge graph contains abundant information with multi-type relations among multi-type nodes. The heterogeneous structure reveals not only the connectivity but also the complementarity between the nodes within a KG, which helps to capture the signal of potential interest of the user. However, existing research works have limited abilities in dealing with the heterogeneous nature of knowledge graphs, resulting in suboptimal recommendation results. In this paper, we propose a new recommendation method based on iterative heterogeneous graph learning on knowledge graphs (HGKR). By treating a knowledge graph as a heterogeneous graph, HGKR achieves more fine-grained modeling of knowledge graphs for recommendation. Specifically, we incorporate the graph neural networks into the message passing and aggregating of entities within a knowledge graph both at the graph and the semantic level. Furthermore, we designed a knowledge–perceiving item filter based on an attention mechanism to capture the user’s potential interest in their historical preferences for the enhancement of recommendation. Extensive experiments conducted on two datasets in the context of two recommendations reveal the excellence of our proposed method, which outperforms other benchmark models.

## Introduction

The last decade has witnessed a rapid development of deep learning applied in the field of recommendation^1^. However, traditional deep learning recommendation systems have the problem of cold start and fail to take advantage of the deep relationships between users and items. As a result, their recommendation results were disappointing when faced with massive, real-time, heterogeneous data^2^. Some researchers seeking solutions with the intent to alleviate these problems have tried to combine deep learning with some new technologies. In recent years, some excellent knowledge graphs (e.g., DBPedia, wikidata, satori) have attracted increasing attention. These methods of incorporating knowledge graphs into recommendation have gradually entered the vision of researchers in various fields.

**Figure 1:**
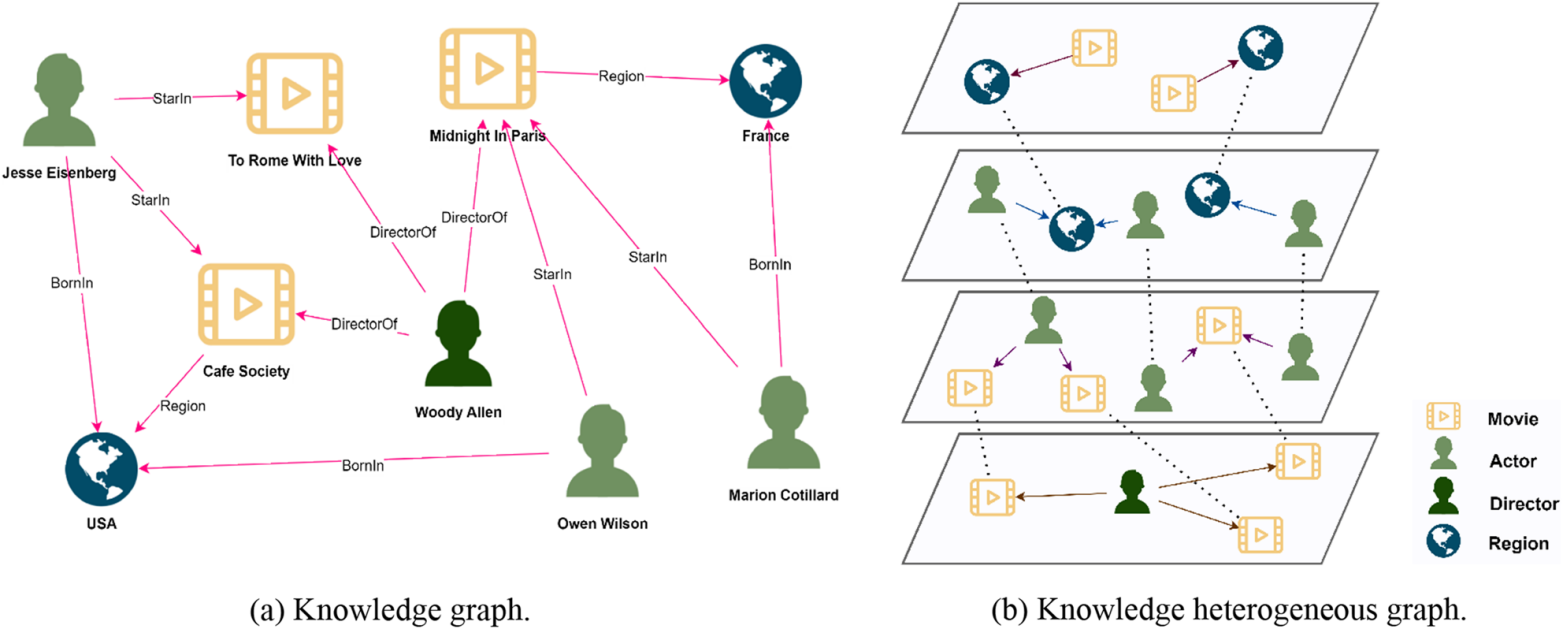
(**a**) describes a knowledge graph constructed on movies and (**b**) shows the bipartite graphs extracted from the knowledge graph of (**a**). Each bipartite graph corresponds to a type of relation in the knowledge graph.

A knowledge graph represents domain knowledge in a special network structure, storing data in the clear and concise form of triples for entities and their interlinks^3^ as shown in Fig. 1a. A knowledge graph is an essentially heterogeneous graph that contains abundant information about different types of nodes and relations. Due to its special structure, the knowledge graph has a great advantage in storing sparse and heterogeneous data, making it able to describe semantic details of entities within a knowledge graph. Focusing on the recommendation task, a lot of research and experiments demonstrate that knowledge graphs can not only help the recommendation algorithms solve the challenge of cold start, but also improve the interpretability and efficiency of recommendation results^4^.

Existing knowledge graph embedding (KGE) methods such as TransE, TransR, and TransD etc.^5–7^, represent the entities and their relations by regularizing them into a vector space and training their embeddings through a unified formula. Although widely used in the field of recommendation, we argue that these methods do not consider the multi-semantic and indirect connections between nodes within a knowledge graph^8^, resulting in limited effectiveness in searching for the potential interest of users.

Previous works have shown great superiority in introducing knowledge graphs into recommendation systems^9^, most of them either utilizeknowledge graphs for embedding representation learning^10–13^, or feature mining using the graph’s unique data structure^14–16^. For example, MKR^10^ designed a multi-task training framework for embedding learning and recommendation tasks, RippleNet^17^ explores users’ potential interests through exploiting the neighborhood of a user’s preferences with an attention mechanism. However, MKR is unreliable in capturing the high-order correlation between entities since the method is partly based on a knowledge graph embedding learning which ignores the path and adjacent information. While RippleNet relies too much on the proportionality of relationships and ignores the integrity of the knowledge graph. Other works relying on KGE, use methods that map users into the knowledge graph^11,14,18^ as nodes, enhancing the efficiency and interpretability of recommendation. Although all these methods have excellent recommendation performance, they still uniformly train the models that have limitations in representing the graphs of multiply nodes and relations. We argue that, in passing messages from different relations, it is insufficient by only differing embeddings. We believe that considering the heterogeneity of the knowledge graph, the information propagation behavior may be different between different nodes of different semantics and different relationships, and how to mode the knowledge graph in a more fine-grained way is an optimization direction.

To address the shortcomings of prior studies, an iterative heterogeneous graph learning recommendation method based on the knowledge graph is proposed in this paper(HGKR). In HGKR, we incorporate the advantages of previous works and adopt a new way of modeling feature learning. Firstly, we extract bipartite graphs from the knowledge graph. As shown in Fig. 1b, all semantic information in a knowledge graph can be represented by four bipartite graphs, each of which records the relationship between two types of entities. Next, we apply graph neural networks to characterize the embedding representation of each node in the bipartite graph at the graph level. Furthermore, we iterate through all the graphs and iteratively propagate messages from one graph to another at the semantic level, which updates the embedding of nodes to retain the semantic information. Besides, we designed a knowledge-perceive filter that utilizes attention mechanism utilized to explore users’ potential interest in their historical preferences. Our HGKR has two advantages over existing methods: (1) Through achieving more fine-grained modeling of the knowledge graph, HGKR can adopt more appropriate message-passing algorithms for different relationships to effectively characterizes a KG’s rich semantic information. (2) The attention weights are calculated on the user’s interaction history, which in turn captures the high-order relatedness of the item, which discovers the collaborative signal for a reasonable recommendation.

The main contributions of this work can be summarized as follows:

- For the recommendation task, we construct knowledge graphs aided by external knowledge base to obtain rich relationships and the attributes of each item. Then we extract bipartite graphs at the semantic level by classifying the triplelt data from the knowledge graph.
- We propose HGKR, an end-to-end framework for recommendation assisted by a knowledge graph. HGKR applies graph neural networks to hierarchical modeling of the knowledge graph, realizing a more fine-grained modeling method.
- Furthermore, we designed a knowledge–perceiving filter to capture the potential interests of users by leveraging the attention mechanism in HGKR.
- We have evaluated the proposed model on two open datasets. Experimental results showed that our proposed method performs better than several state-of-the-art baselines.

The following sections are organized as follows: “Related Work” Section introduces the related work. “Methodology” Section describes the main task description and methodology. “Experiments” Section then introduces the experimental setup, comparative baselines and experimental results. Lastly, “Conclusion and Future Work” Section briefly summarizes the work and gives an outlook.

## Related work

### Knowledge graph embedding

Knowledge graph embedding techniques are wildly applied to guide the representation learning of entities and relations, mapping them into a continuous dimensional vector space^19^.

Bordes et al. proposed TransE^5^ in 2013, which introduced a classical idea based on “translation”, using triplet head entities and relations in a knowledge graph to deduce tail entities based on a translation formula. However, TransE insufficiently dealt with the representation of entities under reflexive and one-to-many relationships in the knowledge graph. To handle these problems, Wang et al. extended the TransE and proposed TransH^20^, constructing a hyperplane corresponding to each relation. Head and tail entities in TransH are mapped to hyperplanes for “translation” learning. Following this idea, TransR^6^, TransD^7^, and TransG^21^ were put forward in succession. Translation models are simple in training and efficient in representing.

However, the methods above only consider a single triplet, they ignore implicit information such as relational paths and the structure of a knowledge graph. Lin et al.^22^ proposed a path-based model PtransE, utilizing a path-constraint resource allocation algorithm to measure the confidence of a relational path and enhance the knowledge reasoning ability. PathCon^23^ considers not only the information of the adjacent edges of entities but also the multiple paths connected with the head and tail entities to acquire information brought by the intermediate entities. InterERP^24^ employs the inception network to increase the interactions between entities and relations to enhance knowledge reasoning ability.

The development of KGE and the increasing demand for knowledge cognition promotes the development of knowledge reasoning and knowledge graph completion^25^. As the underlying part of knowledge graph construction, KGE also plays an important role in the fields that apply knowledge graphs.

### Recommendation system based on knowledge graph

Due to the impressive performance of KGE in knowledge reasoning and completion, more and more research applies KGE in recommendation. MKR^10^ designed a unique feature interaction unit that connects the recommendation task and KGE, realizing joint training between two modules. Inspired by MKR, CAKR^26^ optimizes the feature interaction unit by employing an attention mechanism to enhance reasonable embedding. KTUP^11^ also adopted the method of jointly training recommendation tasks and knowledge graph completion to take users as nodes of knowledge graph for representation learning. Meanwhile, a new KGE method was proposed in KTUP to capture the relationship between users and items by using implicit preferences. Chen et al.^12^ designed a knowledge aware collaborative learning framework, which utilized TransR to learn embedding representations of users and items. Finally, top-k recommendation was made to target users based on calculations using the items’ representation.

Some research mines information between entities focusing on the data structure and connected paths within a knowledge graph for improving the recommenders. RippleNet^17^ is a state-of-the-art work that naturally incorporated a knowledge graph into the recommender system. RippleNet stimulated the propagation of users’ preferred items over the set of linked entities, offering the user’s potential interest. In order to figure out the basic rationale of a user-item interaction, KPRN^14^ conducted knowledge reasoning on paths by leveraging the sequential dependencies within paths connected to users and items and designed a weighted operation for path distribution. Zhang et al.^15^ used graph neural networks for feature extraction of nodes in the graph, for the purpose of preserving higher-order neighborhood information and achieving node-level and graph-level representation of the knowledge graph. Besides, KGCN^16^ and KGAT^27^ focused on the graph structure and employed the attention mechanism in neighbors to mine associated attributes of each node. KGCN sampled from nodes’ neighbors as a receptive field, which models proximity information for each node. While KGAT recursively learns nodes’ embeddings by propagating messages from neighbors.

### Graph neural network

Previous works show that graph neural networks (GNN) are widely applied in recommenders based on knowledge graphs. To model graph structural data^28^, graph neural networks use message passing to aggregate or spread neighborhood features from nodes to nodes. GCN^29^ grafts convolution neural networks to graph structures, implements convolution operations on graph structure data, and aggregates neighbor features for each node in the graph. Instead of using a full size of neighbor set, GraphSAGE^30^ samples a fixed-sized neighbor, and conducts on three different aggregators for the neighbors’ message. GAT^31^ leverages the attention mechanism into a message aggregating step, by computing the hidden states of each node while attending to its neighbors^32^. For heterogeneous graph learning, HAN^33^ designed a novel heterogeneous graph neural network based on hierarchical attention, including node-level and semantic-level attention.

Our proposed recommender model is used to unify the application of graph neural networks in heterogeneous graph learning and recommendation tasks since the graph neural networks are powerful in mining the high-order proximity information between entities in a graph that helps HGKR make better recommendations.

## Methodology

### Background and definition

In the traditional recommendation scenario, the set of *M* users is defined as ${\mathcal{U}} = \left\{ {u_{1} ,u_{2} , \ldots ,u_{M} } \right\}$ and the set of *N* items is defined as ${\mathcal{V}} = \left\{ {v_{1} ,v_{2} , \ldots ,v_{N} } \right\}$. The user-item interaction matrix $Y \in {\mathbb{R}}^{M \times N}$ describes the historical interactions according to the user’s implicit feedback, where $y_{uv} = 1$ indicates that $u$ has a positive engagement with item $v$ (e.g. clicking, watching or browsing), otherwise $y_{uv} = 0$.

A knowledge graph is defined as ${\mathcal{G}} = \left\{ {\left( {h,r,t} \right){|}h, t \in {\mathcal{E}},r \in {\mathcal{R}}} \right\}$. Here $h$, $r$ and $t$ compose the knowledge graph and represent the head, relation, and tail of a knowledge triple respectively. In addition, ${\mathcal{E}}$ denotes the set of entities and ${\mathcal{R}}$ denotes the set of relations.

Knowledge graphs store heterogeneous data as different types of entities and together with their relations, ther are constructed as triplet data. We separate knowledge graph triples into multiple isomorphic bipartite graphs by classifying the relations between nodes. A bipartite graph records the interlinking of the semantic from one set of nodes to another. In addition, we term the bipartite graphs extracted from a knowledge graph as a knowledge heterogeneous graph (KHG).

Given a KG ${\mathcal{G}}$ with $n$ entity types and $r$ relation types, which we have already acquired during the construction of the knowledge graph, the entity set can be divided as ${\mathcal{E}} = \left\{ {{\mathcal{E}}_{1} ,{\mathcal{E}}_{2} , \ldots ,{\mathcal{E}}_{n} } \right\}$, as well as the relation set ${\mathcal{R}} = \left\{ {{\mathcal{R}}_{1} ,{\mathcal{R}}_{2} ,...,{\mathcal{R}}_{r} } \right\}$. Then we classify knowledge graph triples by the relation type where a type of relation ${\mathcal{R}}_{i}$ corresponds to a bipartite graph ${\mathcal{B}}_{i}$ that is composed of head–tail tuples:

1 $${\mathcal{B}}_{i} = \left\{ {\left( {h,t} \right){|}\left( {h,r,t} \right) \in {\mathcal{G}},r \in {\mathcal{R}}_{i} } \right\}$$

where $h \in {\mathcal{E}}_{p}$, $t \in {\mathcal{E}}_{q}$, ${\mathcal{E}}_{p}$ and ${\mathcal{E}}_{p}$ are the subset of ${\mathcal{E}}$, ${\mathcal{E}}_{p} \subseteq {\mathcal{E}},{\mathcal{E}}_{q} \subseteq {\mathcal{E}}$. In this case, ${\mathcal{R}}_{i}$ describes the relationship between two types of entity sets.

Finally, a knowledge heterogeneous graph can be formulated as:

2 $${\mathcal{G}}_{H} = \{{\mathcal{B}}_{i} {|} {\mathcal{R}}_{i} \in R\}$$

where $${\mathcal{G}}_{H} \approx {\mathcal{G}}$$.

Given a user $u$ and a target item $v$ in a typicalrecommendation scenario, our task is to predict whether the user $u$ has any potential interest in item $v$ where $u$ has never had any previous interaction with *v*. The prediction introduces a user-item interaction matrix $Y$ as well as a knowledge heterogeneous graph ${\mathcal{G}}_{H}$ can be formulated as:

3 $$\hat{y}_{uv} = f_{\Theta } \left( {u,v|\Theta ,Y,{\mathcal{G}}_{H} } \right),$$

where $f$ is the prediction function, and ${\Theta }$ represents the parameters of the underlying model.

### Model framework

**Figure 2:**
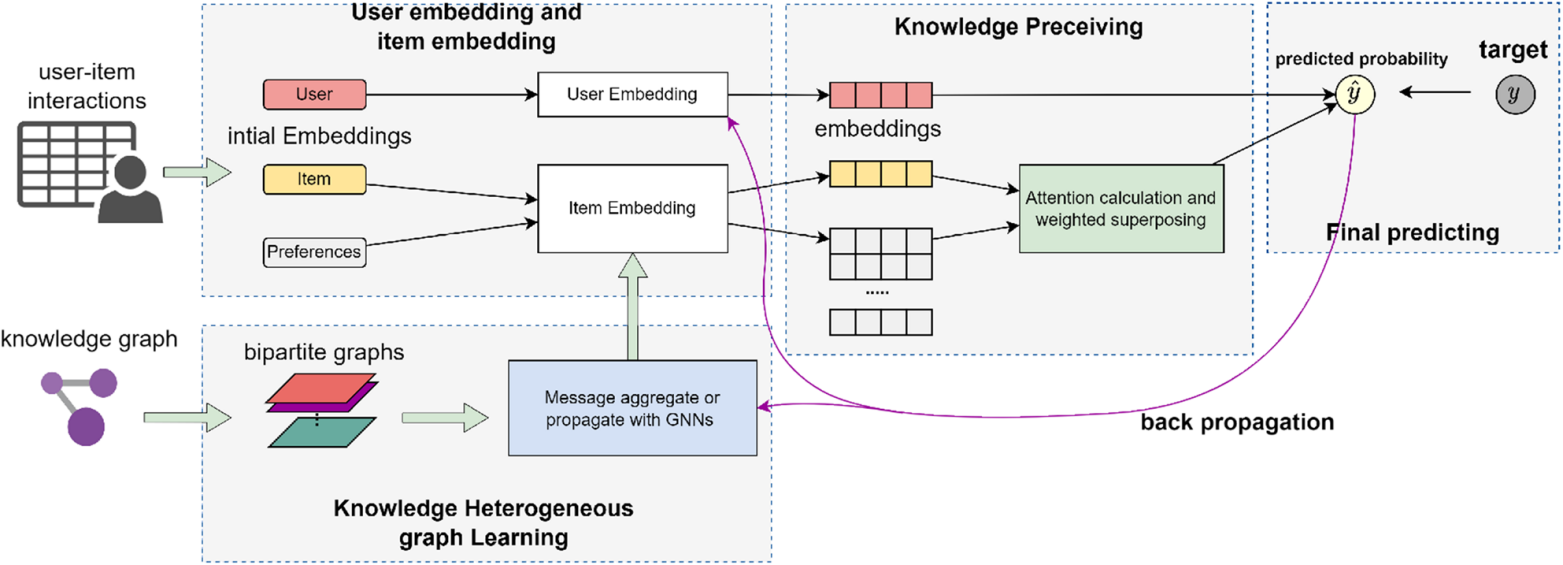
HGKR’s (heterogeneous graph learning on knowledge graph for recommendation) overall framework and its four components. The framework takes user-item interactions and knowledge graph as input and outputs the predicted probability as the recommendation result.

The framework of HGKR is shown in Fig. 2 and its four main components are as follows: (1) Heterogeneous Graph Learning (component)—uses graph neural networks for message passing between nodes in graph level and bipartite graphs at the semantic level. (2) Embedding Module—the module includes a user embedding layer and an item embedding layer trained from heterogeneous graph learning and initializes vector embedding representations of users and items in every training epoch. (3) Knowledge Perceiving Filter—utilizes an attention mechanism to perceive deep relevance between recommended items and users’ preferences, working as collaborative filtering for items. (4) Predicting Module—calculates the user’s final potential-liking score for the target item and the loss of results.

### Heterogeneous graph learning

**Figure 3:**
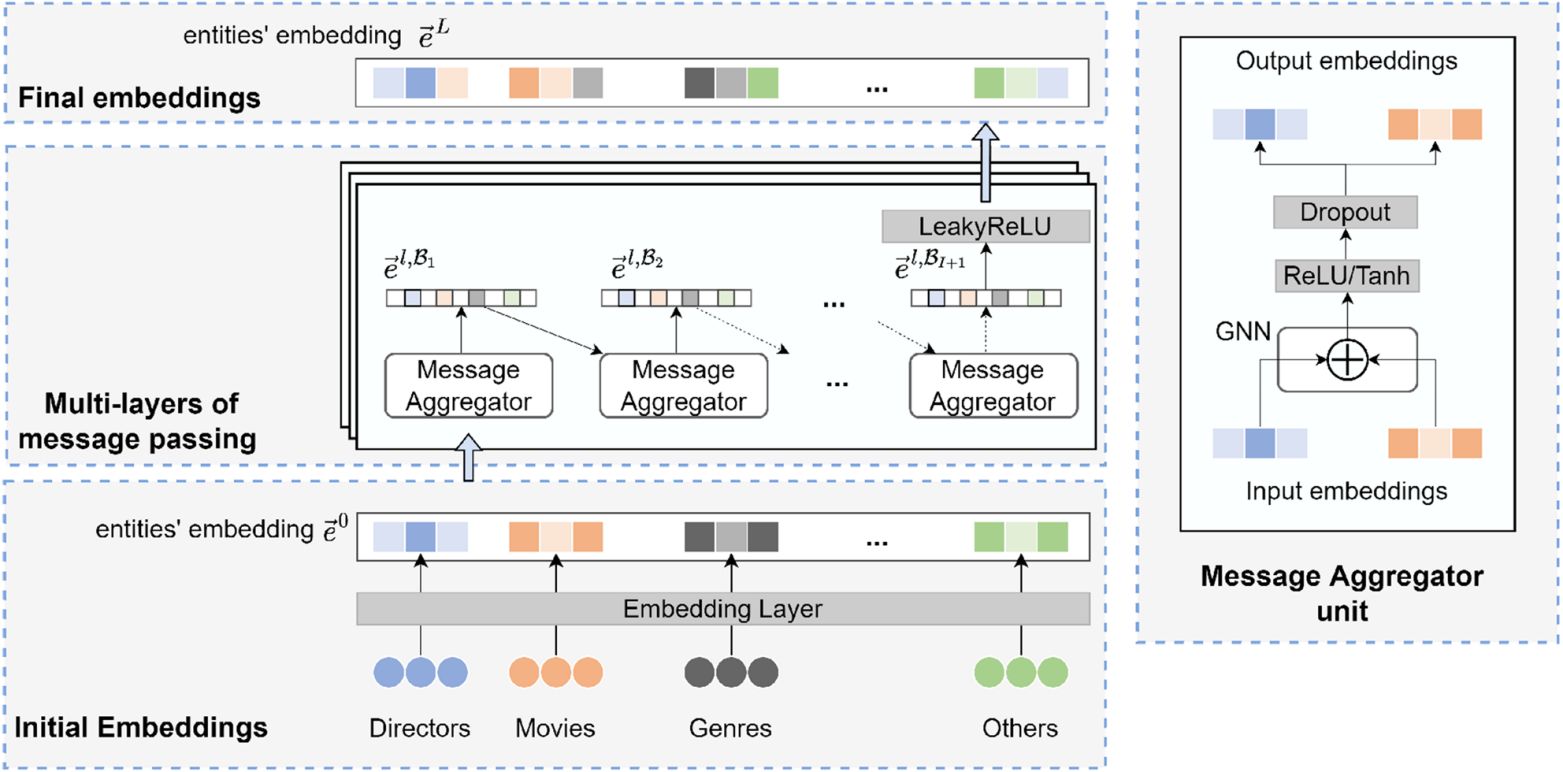
The left part describes the whole process of message passing for heterogeneous graph learning. The right part shows the details of messages aggregator where embeddings of entities are iteratively updated in this module.

In order to mine information with richer and higher-order correlations between entities, we designed a multi-layer message passing module for information communication within a knowledge heterogeneous graph between all bipartite graphs. Technically, at the graph level we apply a graph neural network for nodes to propagate or aggregate features from their neighbors in a bipartite graph. At the semantic level, we run over all bipartite graphs and iteratively update their embeddings. Figure 3 shows the training process for heterogeneous graph learning.

We take the KHG as input into the message passing module where the message aggregator units apply graph neural networks correlation to the bipartite graphs. Assume that we have a KHG with $I$ bipartite graphs ${\mathcal{G}}_{H} = \left\{ {{\mathcal{B}}_{1} , \ldots ,{\mathcal{B}}_{I} } \right\}$, $\forall i \in \left\{ {1, \ldots ,I} \right\}$, a $L$ layer message passing module $\forall l \in \left\{ {1, \ldots ,L} \right\}$, as well as a set of trainable matrices ${\mathbf{W}}^{{L,{\mathcal{B}}_{i} }}$ that assigns weights to features of the node’s embedding as inputs for heterogeneous graph learning. In the learning segment, two GNNs, GAT^31^ and GraphSAGE^30^ are employed as the message aggregator units to conduct the message passing between different bipartite graphs and layers within the model.

Specifically, for a node $z \in {\mathcal{E}}$, we initialize it randomly into vector space as $\vec{e}_{z}^{0}$, where $\vec{e}_{z} \in {\mathbb{R}}^{d}$ is the embedding of the node $z$, and *d* represents the embedding dimension. We iterate over all bipartite graphs in each message-passing layer to aggregate information from a node’s semantical neighbors. The representation in this process of node $z$ can be expressed through:

4 $$\left\{ {\begin{array}{*{20}l} {\vec{e}_{z}^{{l,{\mathcal{B}}_{i + 1} }} = AGG^{{{\mathcal{B}}_{i} }} \left( {\vec{e}_{z}^{{l,{\mathcal{B}}_{i} }} ,{\mathcal{N}}^{{{\mathcal{B}}_{i} }} \left( z \right),{\mathbf{W}}^{{L,{\mathcal{B}}_{i} }} } \right),} \hfill & {{\text{if}}\; z\; {\text{engaged}}\;{\text{in}}\; {\mathcal{B}}_{i} {;}} \hfill \\ {\vec{e}_{z}^{{l,{\mathcal{B}}_{i + 1} }} = \vec{e}_{z}^{{l,{\mathcal{B}}_{i} }} ,} \hfill & {{\text{otherwise}}{.}} \hfill \\ \end{array} } \right.$$

5 $$\begin{array}{*{20}c} {\vec{e}_{z}^{l + 1} = LeakyReLU\left( {\vec{e}_{z}^{{l,{\mathcal{B}}_{I + 1} }} } \right)} \\ \end{array}$$

where ${\mathcal{B}}_{i}$ denotes a bipartite graph we iterate through, and ${\mathcal{N}}^{{{\mathcal{B}}_{i} }} \left( z \right)$ denotes the neighbor information associated with node $z$ in ${\mathcal{B}}_{i}$. Note that if node $z$ is not engaged in the bipartite graph ${\mathcal{B}}_{i}$, the expression of node $z$ should be inherited as $\vec{e}_{z}^{{l,{\mathcal{B}}_{i + 1} }} = \vec{e}_{z}^{{l,{\mathcal{B}}_{i} }}$. $AGG^{{{\mathcal{B}}_{i} }}$ is a differentiable message aggregator function corresponding to a bipartite graph. Furthermore, a *LeakyRuLU* (with negative input slop $\alpha = 0.02$) activation is applied to all nodes before we put their embeddings into the next layer of message passing.

In HGKR, we mainly apply two message passing graph neural networks for the message aggregating function at the graph level: GraphSAGE^30^ and GAT^31^ which means $AGG^{{{\mathcal{B}}_{i} }} \in \left\{ {AGG_{sage} ,AGG_{gat} } \right\}$. Sampling a specific node $z$ in a bipartite graph, the aggregator algorithm with GNN details is as follows:

- *GraphSAGE* In the GraphSAGE aggregator, which is a mean operator, we simply aggregate the elementwise mean of the vectors in $\left\{ {\vec{e}_{k} ,\forall w \in {\mathcal{N}}\left( z \right)} \right\}$. The aggregator function $AGG_{sage}$ is formulated as:
  6 $$\vec{e}_{{{\mathcal{N}}\left( z \right)}} = MEAN\left( {\left\{ {\vec{e}_{k} ,\forall k \in {\mathcal{N}}\left( z \right)} \right\}} \right)$$
  7 $$AGG_{sage} = \sigma \left( {{\mathbf{W}}_{{{\varvec{sage}}}} \cdot CONCAT(\vec{e}_{z} ,\vec{e}_{{{\mathcal{N}}\left( z \right)}} )} \right)$$

where ${\mathbf{W}}_{{{\varvec{sage}}}} \in {\mathbb{R}}^{{d^{\prime } \times 2d}}$ is a linear transformation parameterized by a weight matrix and $d^{\prime }$ is the hidden dimension of the model. $CONCAT$ denotes the vector concatenation operation and $\sigma$ is a non-linearity activation.

- *Graph Attention Network* The GAT aggregator employs the attention mechanism for aggregating neighborhood information. For vectors in $\overrightarrow {{\{ e}}_{k} ,\forall k \in {\mathcal{N}}\left( z \right)\}$ we compute the attention coefficient as:
  8 $$\pi_{zk} = {\vec{\text{a}}}^{T} \left[ {CONCAT\left( {{\mathbf{W}}_{{{\varvec{gat}}}} \vec{e}_{z} ,{\mathbf{W}}_{{{\varvec{gat}}}} \vec{e}_{k} } \right)} \right]$$
  9 $$\alpha_{zk} = \frac{{{\text{exp}}\left( {LeakyReLU\left( {\pi_{zk} } \right)} \right)}}{{\mathop \sum \nolimits_{{w \in {\mathcal{N}}\left( z \right)}} {\text{exp}}\left( {LeakyReLU\left( {\pi_{zw} } \right)} \right)}}$$
  where ${\vec{\text{a}}}^{T}$ denotes a single-layer feedforward neural network, parameterized by a weight vector ${\vec{\text{a}}}$, and $\cdot^{T}$ represents a transposition operation. ${\mathbf{W}}_{{{\varvec{gat}}}} \in {\mathbb{R}}^{{d^{\prime } }}$ is a learnable linear transformation to transform features into higher-level features for getting sufficient expressive ability.

In addition, the aggregated features from neighborhood information are weighted and summed to obtain $\vec{e}_{z}$. The aggregator function $AGG_{gat}$ can be expressed with a non-linearity $\sigma$:

10 $$AGG_{gat} = \sigma \left( {\mathop \sum \limits_{{w \in {\mathcal{N}}\left( z \right)}} \alpha_{zw} {\mathbf{W}}_{{{\varvec{gat}}}} \vec{e}_{w} } \right)$$

Experimentally, in HGKR we apply the GAT aggregator only to the bipartite graphs tailed with recommendation item type of entities for message aggregation, while GraphSAGE aggregator is applied to the rest of the case.

### Knowledge-perceive filter and prediction

In order to find out whether a user is interested in a particular item, we search for the answer using the user’s past interactions. We regard a user’s past interactions as positive feedback for the user’s preferences. For a user $u$ and their historical interactions $H\left( u \right)$, the user preferences set is expressed as:

11 $${\mathcal{P}}_{u} = \{ p\sim H\left( u \right)|y_{up} = 1\}$$

Assume that we want to recommend an item $v$ to a user $u$, we calculate the correlation coefficient by using the inner product between $v$ and each item in ${\mathcal{P}}_{u}$ as:

12 $$\beta_{vk} = softmax\left( {\vec{e}_{v}^{T} \cdot \vec{e}_{k} } \right) = \frac{{\exp \left( {\vec{e}_{v}^{T} \cdot \vec{e}_{k} } \right)}}{{\mathop \sum \nolimits_{{p \in {\mathcal{P}}_{u} }} \exp \left( {\vec{e}_{v}^{T} \cdot \vec{e}_{p} } \right)}}$$

**Figure 4:**
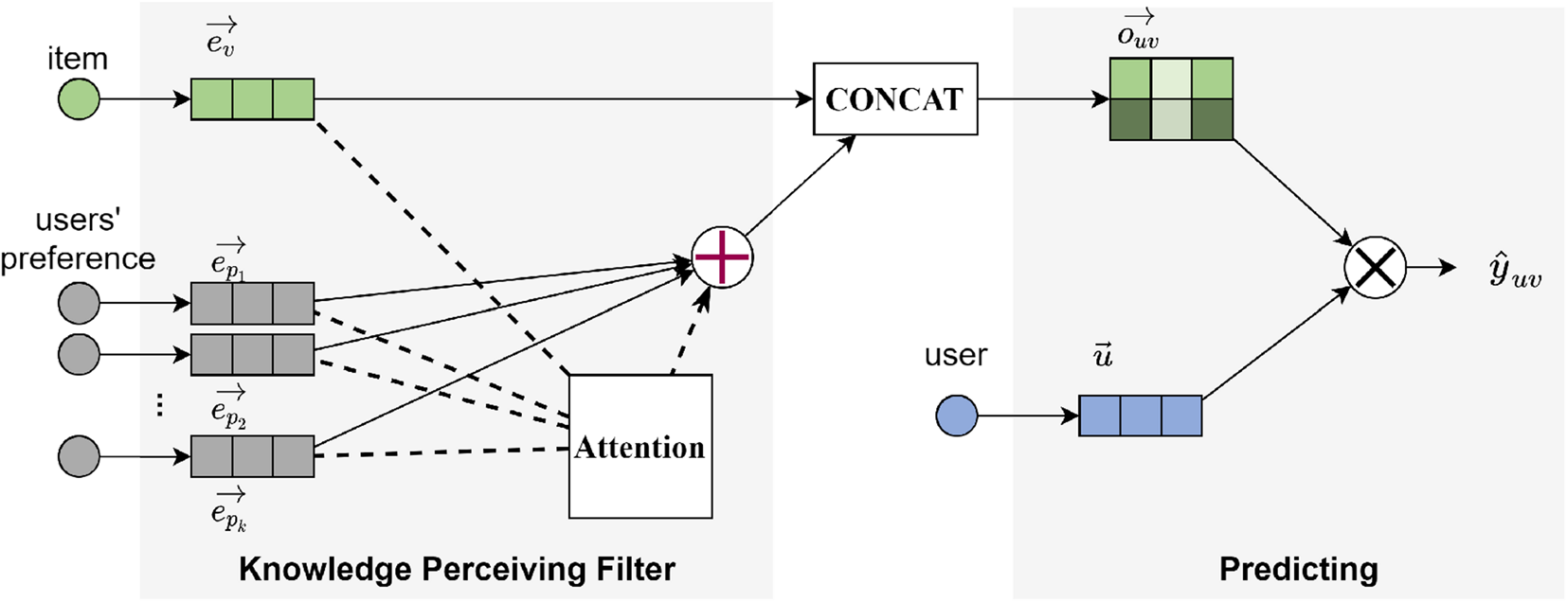
The process of knowledge perceiving and final predicting. The left part of knowledge perceiving filter calculates attention weight in users’ preferences. The right part simply conducts a cross-product to determine the final probability scores.

Next, we weight and superpose preferences items and concatenate the result $\vec{h}_{uv}$ onto the target item $\vec{e}_{v}$. The item-preferences relevance vector $\vec{o}_{uv}$ can then be obtained by:

13 $$\vec{h}_{uv} = \sigma \left( {\mathop \sum \limits_{{p \in {\mathcal{P}}_{u} }} \beta_{vp} \vec{e}_{p} } \right)$$

14 $$\vec{o}_{uv} = {\mathbf{W}}_{o} \cdot CONCAT\left( {\vec{e}_{v} ,\vec{h}_{uv} } \right) + b$$

where ${\mathbf{W}}_{o} \in {\mathbb{R}}^{d \times 2d}$ is a trainable linear transformation followed by a bias ${\text{b}}$, ${\text{b}} \in {\mathbb{R}}^{d \times d}$. Figure 4 shows the process of knowledge perceiving and predicting the final scores.

After capturing the relevance for item $v$ in user preferences, we predict the user’s potential preferences by taking the cross product of the user embedding and the output of the knowledge perceiving item filter:

15 $$\hat{y}_{uv} = \phi \left( {\vec{e}_{u} \times \vec{o}_{uv} } \right)$$

$\phi \left( \cdot \right)$ represents the *sigmoid* function.

### Learning algorithm

In HGKR, we treat the recommendation task as a binary classification problem, so we employ the binary cross-entropy loss between the ground truth of user-item interactions ${\mathcal{Y}}$ and the predicted value to indicate our training process. The complete loss function is as follows:

16 $$\begin{aligned} L & = {\mathcal{L}}_{bce} + {\mathcal{L}}_{L2} + {\mathcal{L}}_{AGG} \\ & = \mathop \sum \limits_{{\left( {u,v} \right) \in {\mathcal{Y}}}} \left( { - \left( {{\text{y}}_{uv} {\text{log(}}\hat{y}_{uv} {)} + \left( {1 - {\text{y}}_{uv} } \right){\text{log(}}1 - \hat{y}_{uv} {)}} \right)} \right) \\ & \quad + \lambda_{1} \mathop \sum \limits_{e \in \varepsilon } \parallel E\parallel_{2}^{2} + \lambda_{2} \mathop \sum \limits_{r \in R} (\parallel {\mathbf{W}}_{{\text{r}}} \parallel_{2}^{2} + \parallel b_{r} \parallel_{2}^{2} ) \\ \end{aligned}$$

In the above function, $E$ represents the embedding matrices for all entities in KHG. The second term ${\mathcal{L}}_{L2}$ is the L2-regularization of embeddings for preventing over-fitting. Then the third term ${\mathcal{L}}_{AGG}$ is the regularization for GNNs in message aggregators. Finally, $\lambda_{1}$ and $\lambda_{2}$ are the regularization weights.

To alleviate the sparse gradient problem, we employ the Adam stochastic optimization^34^ to optimize the loss ${\mathcal{L}}$ of our model iteratively. Next, we apply a training method of randomly sampling a mini batch of interactions from $Y$, followed by a back-propagation on the sampled mini batch to update model parameters ${\Theta }$ in order to make the training process more effective and efficient.

The overall forward algorithm of HGKR is shown in Algorithm 1.
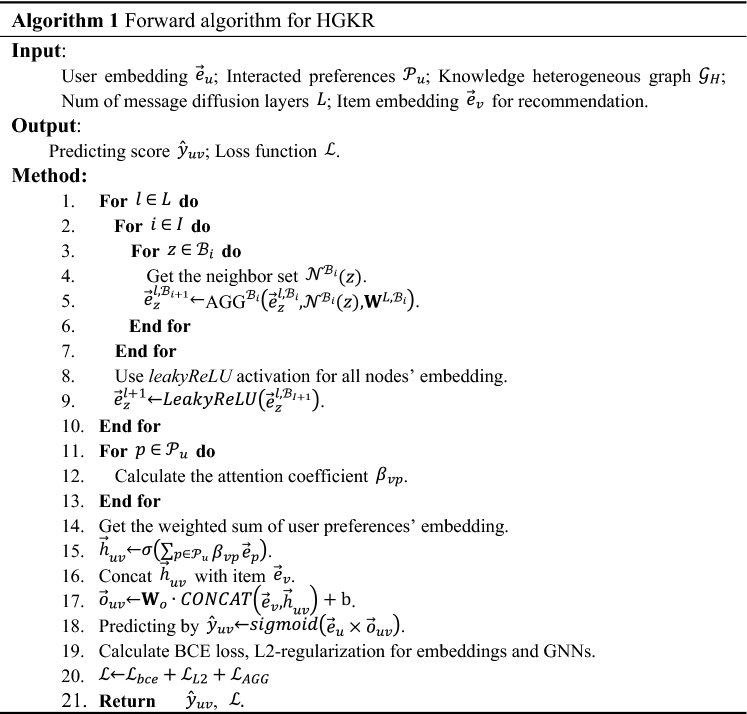


## Experiments

In this section, we evaluate the performance of our proposed model HGKR on two real recommendation scenarios: movies and MOOC.

### Dataset description

The datasets we consider in our experiments are MovieLens Latest and MOOCCube.

*MovieLens Latest* A widely used benchmark dataset in the field of movie recommendations which consists of approximately ten hundred thousand explicit ratings ranging from 1 to 5. In our model, a binary-classification task, we consider the ratings above 3 as positive interactions between users and movies and the ratings of 3 or below as negative.

*MOOCCube* A Chinese massive open online courses dataset that contains about 2 million user-course participation records. In this experiment we set the feedback as positive if a user has had an interaction with a course and negative otherwise.

To construct KG and KHG for ml-latest, we obtain auxiliary information on movies using Wikidata and group the data into triples. MOOCCube already contains abundant descriptions about a course (e.g. concept, lecturer,school, etc.) so we do not need to use an additional data source in this case. Some statistical details are shown in Table 1.

**Table 1:** Statistics of ml-latest and MOOCCube.

Classification	ml-latest	MOOCCube
User-item interactions		
# users	610	5044
# items	9742	705
# interactions	100,836	368,484
Knowledge graph		
# entities	51,601	3,526
# relations	6	6
# triples	180,353	16,874
Knowledge heterogeneous graph		
# entity types	7	5
# relation types	6	6
# bipartite graphs	6	6

For each dataset, we split and randomly select 75%, 15% and 15% of user history interaction data as the train, validate and test set respectively.

### Baselines

We compare our proposed method with five state-of-art methods. Details are listed as follows:

- NFM^35^ is a state-of-the-art factorization model in CTR scenarios. We extract sparse features of items from the knowledge graph and model the second-order feature interactions using NFM.
- CKE^13^ combines various entities’ embeddings from different backgrounds for a unified recommendation framework. We implement CKE with a structural knowledge embedding learning TransR^6^ in this paper.
- MKR^10^ is a generalized framework over several representative methods of knowledge graph embedding task and recommendation task via cross and compress units which share high-order interactions between two tasks.
- KGCN^16^ captures and aggregates items’ associated attributes by sampling from their neighbors as their receptive field to determine inter-item relatedness. It takes advantage of GNNs and factorization methods for recommendation.
- RippleNet^17^ is an end-to-end framework that stimulates the propagation of user preferences knowledge entities in the knowledge graph by combining path-based methods and an attention mechanism.
- KGAT^27^ exploits high-order connectivity between items by extracting paths or implicitly modeling them with regularization. KGAT employs the GNNs and the attention mechanism to discriminate the importance of the neighbors of nodes in a KG.

### Evaluation metrics

We conduct HGKR in two typical recommendation scenarios: (1) In click-through rate (CTR) prediction, we employ *AUC* and *Recall* to evaluate the overall performance of our proposed model. (2) In top-*K* recommendation, we employ *Precision@K* and *nDCG@K* to measure the relative order within the highest top-*K* of the predicted items list, where *K* takes values from 1 to 15. In order to eliminate possible errors in the experiment, each experiment is conducted 16 times with the same number of 100 training epochs and we take the average scores as the final results.

### Experiments Setting

To facilitate the comparison of experiments, we set the same number of training epochs as 100 for all models optimized with Adaptive Moment Estimation (Adam), and the same batch size range within {512,1024}, as well as the same learning rate of 0.01. For loss calculation, we set regularization weight $\lambda_{1} ,\lambda_{2}$ as 10^−7^, 10^−4^ respectively. Specifically, for MKR, CKE, KGCN and KGAT, the recommendation embedding dimensions of users and items of ml-latest and MOOCCube are 16, 12, as well as the entities and relations in KGE. The interval of the KGE task in MKR is 3. For RippleNet, the dimension of relations is the square of entities and the number of hops is 4. The sample sizes of neighbors in KGCN and users’ preferences in RippleNet are both set to 32. For NFM, the channels and the number of hidden linears are set to 32 and 3 respectively. For KGAT, the knowledge graph batch size is set as 2024, and the aggregation type is set as bi-interaction as default. In our proposed model HGKR, we set the dimension of entities the same as that of formal experiments where the channels and number of hidden linears are set as 24 and 2, respectively. The size of sampling in users’ preferences $K$ is set to 32 as well. Other hyper-parameters are tuned based on their performance on the validation set.

### Performance comparison

**Figure 5:**
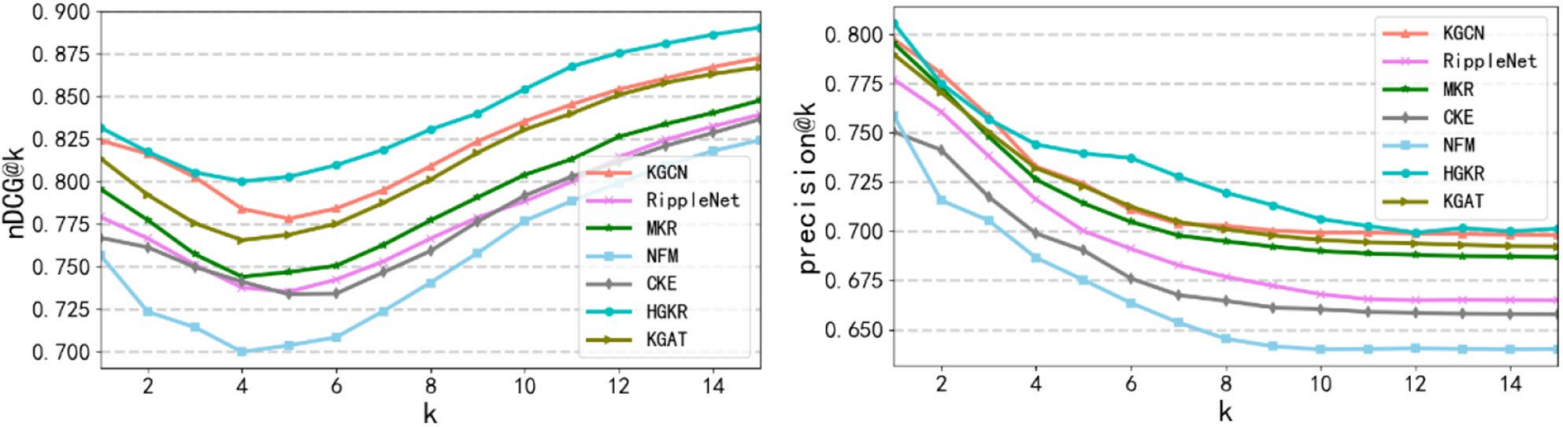
*Precision@K* and *nDCG@K* curves on MOOCCube.

**Figure 6:**
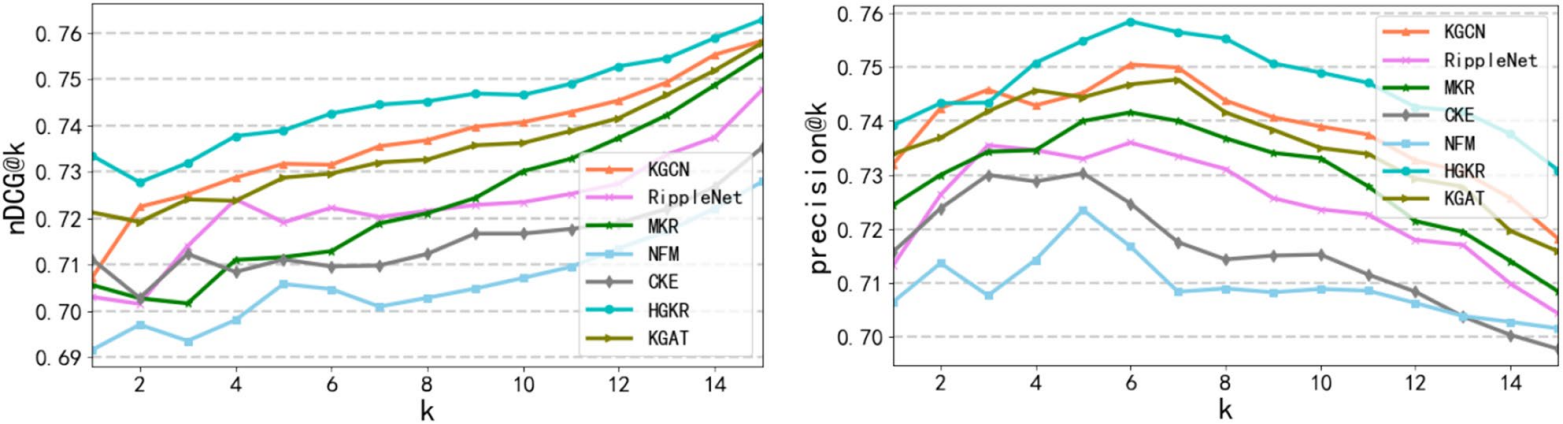
*Precision@K* and *nDCG@K* curves on ml-latest.

In this section, Table 1 shows the baseline performance on two datasets in CTR prediction while Figs. 5 and 6 show *nDCG@K* and *precision@K* curves in top-K recommendation. From our experiments our findings are as follows:

- NFM gives the worst performance among all compared experiments since NFM is the only KG-free model and fails to fully explore the connectivity between entities or users and items.
- CKE simply introduces a KG representation learning to achieving better performance than NFM. Compared with CKE, cross and compress units of MKR make further use of the KG representation learning, making MKR perform better.
- RippleNet performs very well because of its special mechanism of users’ preferences aggregating actual benefits to the recommendation. However, RippleNet shows more instability on ml-latest in top-K recommendation, probably because it relies heavily on the proportionality of relations between entities that are sparser in ml-latest.
- KGCN gives the top performance in all of the baseline experiments. Note that both KGCN and RippleNet mine information on multi-hop neighbors, consistently demonstrating the importance of capturing proximity features at the graph level of a KG for the enhancement of recommendation.
- KGAT performs only slightly second to KGCN. A possible reason is that the embedding aggregation strategy of KGAT is too complicated and brings some noise compared with KGCN under our datasets. The results also demonstrate the advantage of GNNs in modeling the graph structure data in the field of recommendation.

HGKR takes advantage of aggregating neighbors’ information and getting attention of users’ preferences, which comes from the priorities of previous approaches. In addition, the employment of heterogeneous graph representation learning helps HGKR to explore the further connectivity between entities at the semantic level.

As clearly seen from the statistics in Table 2, HGKR outperforms the best in baselines by 0.92% on *AUC* and 0.37% on *Recall* in ml-latest, and by 2.21% on *AUC* and 2.06% on *Recall* in MOOCube data. Then as illustrated in the *top-K* curves in Figs. 5 and 6, HGKR shows superior performance compared to all baselines. From our experiments and analysis we have the following observations:

- HGKR performs more enhancements in MOOCCube than on ml-latest, demonstrating that HGKR can be more powerful in data-dense recommendation scenarios since the semantic information of ml-latest is much sparser than MOOCube.
- The curve of HGKR in *precision@K* of HGKR shows some fluctuations in two datasets. These fluctuations illustrate that HGKR may not the best choice in top2 or 3 recommendation, but is much more capable in top 6 and 7 recommendation. Furthermore the *nDCG@K* curves verify the excellent capacity of HGKR in most cases.

**Table 2:** performance of *AUC* and *Recall* in CTR prediction.

	ml-latest		MOOCCube	
	AUC	Recall	AUC	Recall
NFM	0.7075	0.8538	0.8541	0.6612
CKE	0.7269	0.8576	0.8649	0.6723
MKR	0.7631	0.8617	0.8758	0.6857
KGCN	0.7692	0.8596	0.8912	0.7227
RippleNet	0.7314	0.8484	0.8655	0.6810
KGAT	0.7683	0.8527	0.8882	0.7202
HGKR	0.7763	0.8649	0.9109	0.7376
%Improvement	0.92%	0.37%	2.21%	2.06%

### Ablation study

In this section we compare the influence of different message aggregators on HGKR. Below we use HGKR_Sage_ to denote the model with GraphSAGE for all the message aggregators. Similarly, HGKR_GAT_ denotes the model with all GAT, HGKR_GCN_ denotes the model with all GCN. HGKR employs GAT for message aggregation of item sort of entities and GraphSAGE in the other cases.

Results in Table 3 verify the superior nature of the mixture of GraphSAGE and GAT aggregators. This result can be explained clearly since when aggregating items’ neighbor information we should focus on the highly relevant neighbor entities. Then when dealing with other entities, neighbors should be treated equally to reduce prejudice and noise.  In all cases, GCN performs worse experiments as an information aggregation algorithm than GraphSAGE, probably because GraphSAGE focuses on modeling how information propagates, while GCN simply adds feature vectors.

**Table 3:** Comparison results of ablation experiments.

	ml-latest		MOOCCube	
	AUC	nDCG@10	AUC	nDCG@10
HGKR_SAGE_	0.7592	0.7353	0.8847	0.8428
HGKR_GAT_	0.7701	0.7473	0.9056	0.8491
HGKR_GCN_	0.7526	0.7219	0.8811	0.8407
HGKR	0.7763	0.7477	0.9109	0.8628

To study the parameter sensitivity of HGKR, we focus on the layers’ num $L$ of message passing and the sampling size of users’ preferences as shown by the *AUC* scores in Tables 4 and 5 below. Our findings are as follows:

- The structure of too many layers of message passing module may lead to overfitting. As we can see in Table 3 the scores drop rapidly as $L$ increases, while the $L$ of 2 is the most suitable for HGKR.
- Table 4 indicates that the best sampling size $K$ for HGKR is located between 24 and 32. This is probably because on the one hand too small of a $K$ cannot capture enough attention and relatedness between entities, while on the other hand too large of a $K$ brings significant noise. In the sparser dataset scenario, appropriately reducing $K$ can have an effect of making HGKR perform better.

**Table 4:** *AUC* scores with layers’ num of message passing module.

Layer num $L$	1	2	3	4	5
ml-latest	0.7668	0.7689	0.7641	0.7613	0.7608
MOOCCube	0.9007	0.9108	0.8912	0.8815	0.8811

**Table 5:** *AUC* scores with sampling size of users’ preferences.

Sampling size $K$	8	16	24	32	40	48
ml-latest	0.7605	0.7705	0.7763	0.7682	0.7573	0.7594
MOOCCube	0.8810	0.8901	0.8940	0.9105	0.9027	0.8809

## Conclusion and future work

In this paper, we propose a new framework HGKR that achieves a more fine-grained modeling of knowledge graphs for recommendation. In consideration of the heterogeneity, we extract bipartite graphs from the knowledge graphs and then utilize graph neural networks to iteratively propagate information between nodes at the graph level, and between bipartite graphs at the semantic level. In addition, we designed a knowledge perceiving filter based on an attention mechanism to explore the user’s potential interest and then provide recommendations. The experimental results conducted on two datasets of two scenarios have shown the great effectiveness of our model.

In the future, we will extend our work beyond two limitations. (1) In this work we manually matched graph neural networks with bipartite graphs. Making the process of matching automatic and formulaic is a promising direction. (2) Experiments show the instability of our model when facing the challenge of the sparser dataset. It is worth verifying whether introducing outstanding KG reasoning and completing techniques into our method will be helpful in improving the recommendation.

## Data Availability

The datasets generated and analysed during the current study are available in the mooccube repository, http://moocdata.cn/data/MOOCCube, and movielens repository, https://grouplens.org/datasets/movielens/.

## List of symbols

${\mathcal{U}}$: The set of users
${\mathcal{V}}$: The set of items
$Y$: The user-item interaction matrix
${\mathcal{G}}$: The knowledge graph
${\mathcal{E}}$: The set of entities of a knowledge graph
${\mathcal{R}}$: The set of relations of a knowledge graph
${\mathcal{B}}_{i}$: The bipartite graph of a relation
${\mathcal{G}}_{H}$: The knowledge heterogeneous graph
$\hat{y}_{uv}$: The likely score of a user $u$ to item $v$
$f\left( \cdot \right)$: The prediction function
$\vec{e}_{z}$: The embedding of a node $z$
$AGG^{{{\mathcal{B}}_{i} }} \left( \cdot \right)$: The message aggregator function
${\mathcal{N}}\left( z \right)$: The neighbor information associated with node $z$
$LeakyReLU\left( \cdot \right)$: The LeakyReLU activation
${\mathbf{W}}^{{L,{\mathcal{B}}_{i} }}$: The trainable matrices assign weights to nodes
$p\sim H\left( u \right)$: The set of $u$’s history interactions
${\mathcal{P}}_{u}$: The set of $u$’s historical preferences
$\beta_{vk}$: The correlation coefficient of vectors $v$ and $k$
$\vec{h}_{uv}$: The superposed result of user’s preferences items
$CONCAT\left( \cdot \right)$: The concatenate operation
$\vec{o}_{uv}$: The item-preferences relevance vector
$\phi \left( \cdot \right)$: The sigmoid function
${\mathcal{L}}$: The loss function
